# Rab27A mediated by NF-κB promotes the stemness of colon cancer cells via up-regulation of cytokine secretion

**DOI:** 10.18632/oncotarget.11454

**Published:** 2016-08-20

**Authors:** Feixue Feng, Yinghao Jiang, Huanyu Lu, Xiaozhao Lu, Shan Wang, Lifeng Wang, Mengying Wei, Wei Lu, Zhichao Du, Zichen Ye, Guodong Yang, Fang Yuan, Yanxia Ma, Xiaoying Lei, Zifan Lu

**Affiliations:** ^1^ The State Key Laboratory of Cancer Biology, Department of Pharmacogenomics, Fourth Military Medical University, Xi'an, China; ^2^ Department of Clinical Laboratory, the Affiliated Hospital of Shaanxi University of Chinese Medicine, Xianyang, China; ^3^ Department of Occupational and Environmental Health, the Ministry of Education Key Lab of Hazard Assessment and Control in Special Operational Environment, School of Public Health, Fourth Military Medical University, Xi'an, China; ^4^ The State Key Laboratory of Cancer Biology, Department of Biochemistry and Molecular Biology, Fourth Military Medical University, Xi'an, China

**Keywords:** Rab27A, colon cancer stem cells, NF-κB, secretion of cytokines, microenvironment

## Abstract

Recent evidences have unveiled critical roles of cancer stem cells (CSCs) in tumorigenicity, but how interactions between CSC and tumor environments help maintain CSC initiation remains obscure. The small GTPases Rab27A regulates autocrine and paracrine cytokines by monitoring exocytosis of extracellular vesicles, and is reported to promote certain tumor progression. We observe that overexpression of Rab27A increased sphere formation efficiency (SFE) by increasing the proportion of CD44^+^ and PKH26^high^ cells in HT29 cell lines, and accelerating the growth of colosphere with higher percentage of cells at S phase. Mechanism study revealed that the supernatant derived from HT29 sphere after Rab27A overexpression was able to expand sphere numbers with elevated secretion of VEGF and TGF-β. In tumor implanting nude mice model, tumor initiation rates and tumor sizes were enhanced by Rab27A with obvious angiogenesis. As a contrast, knocking down Rab27A impaired the above effects. More importantly, the correlation between higher p65 level and Rab27A in colon sphere was detected, p65 was sufficient to induce up-regulation of Rab27A and a functional NF-κB binding site in the Rab27A promoter was demonstrated. Altogether, our findings reveal a unique mechanism that tumor environment related NF-κB signaling promotes various colon cancer stem cells (cCSCs) properties via an amplified paracrine mechanism regulated by higher Rab27A level.

## INTRODUCTION

The cancer stem cell hypothesis suggests that many cancers are maintained in a hierarchical fashion, consisting of rare, slowly dividing cancer stem cells (CSCs) or tumor-initiating cells (T-ICs), rapidly dividing amplifying cells or early precursor cells (EPC) and differentiated tumor cells [1–3]. CSCs are self-renewable, able to differentiate into multiple cell lineages, and highly tumorigenic in immunodeficiency mice. CSCs are not only the source of the tumor but also may be responsible for tumor progression, metastasis, resistance to therapy, and subsequent tumor recurrence [1, 2, 4, 5]. Tentatively defined CSCs have been identified in hematologic [6], brain [7], breast [4], prostate [8], liver [9], pancreas [5], and colon cancers [10]. According to the hypothesis, CSCs can be enriched by sorting for stem markers, such as CD24, CD44, CD133 *et al* [4, 7, 11, 12], by selecting for side-population (SP) cells that efflux Hoechst dyes [13, 14] or enhanced PKH26 dye-retaining capacity [15–17], or by isolating spherical clusters of self-replicating colospheres cells from suspension cultures [18, 19].

Colon cancer stem cells (cCSCs) are located in a “niche” made up of fibroblasts, immune cells, endothelia and gliocytes [20]. Although genetic mutations of cCSCs is a key component in tumor progression (APC or Wnt/β-catenin) [21], inflammatory cytokines within the microenvironment affect cCSCs through activation of related pathways such as Notch, Hedgehog, STAT3 and NF-κB [22–24]. Recent studies have reported the pivotal role of NF-κB signaling pathway in the regulation of colorectal and colitis-associated tumorigenesis [25]. Schwitalla *et al* found that elevated NF-kB signaling enhances Wnt activation and induces dedifferentiation of non-stem cells that acquire tumor-initiating capacity [23]. Besides the direct effects of inflammation on CSC initiation, it will be interesting to know whether it can facilitate a cross-talk between CSC and its nearby environment.

Rab proteins are small GTPases belonging to the Ras superfamily and are mainly involved in intracellular vesicle transport [26, 27]. Rab27 is the key protein for intracellular secretion, and contains two isoforms: Rab27A and Rab27B. Dysfunction of Rab27A causes a disorder of melanosome transport called as Griscelli syndrome (GS) [28] and impairs glucose tolerance [29]. Recently, Rab27A was also reported to promote tumor progression [30, 31]. Overexpression of Rab27A promotes growth and metastasis of breast cancer [32] and melanoma [33] in an exosome-dependent or independent manner [34]. The essential features of cancer stem cell are its strong ability of tumor initiation and metastasis. Putatively Rab27A mediated autocrine and paracrine effects may facilitate the cross-talk between CSC and environment. Therefore this study was undertaken to delineate the role of Rab27A in colon cancer, especially its influences on cCSCs and its underlying mechanism, as well as its relevance with NF-κB related inflammatory signal pathway.

## RESULTS

### Rab27A improved cCSC self-renewal *in vitro*

**Figure 1:**
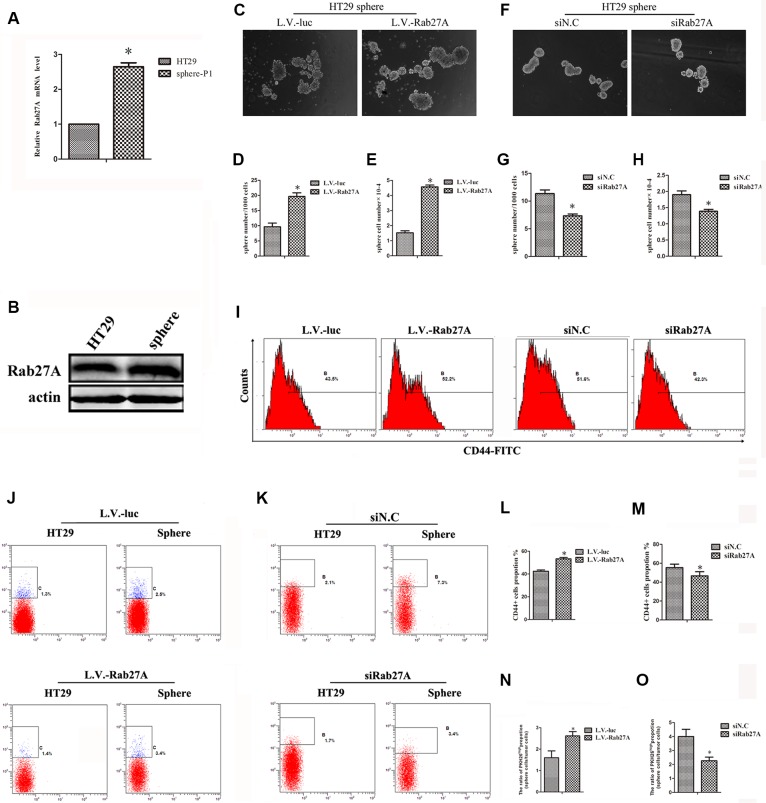
Rab27A improved cCSC self-renewal *in vitro* (**A**) qRT-PCR and (**B**) Western blot showing Rab27A expression was higher in sphere-P1 cells than adherent cells of HT29. Error bars denote the SD between triplicates (*p < 0.01). (**C** and **F**) Representative images of spheres after ectopic Rab27A expression (L.V.-Rab27A, and L.V.-luc was control, C) and Rab27A knockdown (siN.C was scramble control, F). (**D** and **G**) The number of sphere after ectopic Rab27A expression (D) and Rab27A knockdown (G). (**E** and **H**) The number of sphere cells after ectopic Rab27A expression (F) and Rab27A knockdown. (**I**) FACS showing CD44^+^ levels after ectopic Rab27A expression and knockdown. (**J** and **K**) FACS showed the propotion of PKH26^high^ population after ectopic Rab27A expression (J) and knockdown (K). (**L** and **M**) Data of (I) were calculated as mean ± SD as detailed in Materials and Methods. Error bars denote the SD between triplicates (*p < 0.05). (**N** and **O**) Data of (J and K) were calculated as mean ± SD as detailed in Materials and Methods.

In this study, we cultured spheres of six colon cancer cell lines (Supplementary Figure S1) and examined the stemness markers and differentiation markers of HT29 sphere (Supplementary Figure S2A–S2C). We determined that sphere culture could screen and enrich cCSCs. We first found that the expression of Rab27A was enhanced in HT29 sphere cells compared with the adherent state (Figure 1A and 1B). At the same time, we have observed that Rab27A was retained a higher level similar as other stem cell markers (Supplementary Figure S2D). The data above prompted that Rab27A might play a role in the cCSCs. So we explored the function of Rab27A in cCSCs. HT29 cell lines with an overexpression of Rab27A (L.V.-Rab27A) were set up to investigate the role of Rab27A in colon cancer stem cells (Supplementary Figure S3). Compared with control (lentiviral-luciferase–treated cells [L.V.-luc]), forced Rab27A overexpression resulted in a significant increase in the number and size of HT29 spheroids (Figure 1C–1E). We designed two siRNA sequences targeting Rab27A (Supplementary Figure S3). Better performed siRNA was selected for subsequent experiments. After knocking down Rab27A with RNAi, we noticed significant decrease in sphere numbers and shrinkage in sphere sizes (Figure 1F–1H). These data suggested that Rab27A may facilitate cancer development via the expanded self-renewal ability of cCSC.

### Rab27A overexpression improved the proportion of stem cells

In order to validate the above results, CD44+ proportion, which is known as one of the stemness markers in colon cancer, was determined by FACS. We observed that CD44+ proportion was elevated in sphere cells, comparing with HT29 adherent cells (Supplementary Figure S4). This evidence demonstrated that ectopic expression of Rab27A tended to raise CD44^+^ cells compositions in sphere culture, in contrast, the ratio of CD44^+^ declined when Rab27A expression was reduced (Figure 1I, 1L and 1M).

PKH26 is a dye which binds membrane phospholipids, and was used to label stem cells which were selected from the PKH26^high^ population through FACS ^[15–17]^. We observed that the ratio of PKH26^high^ population in colon sphere condition was higher than adherent HT29 cells (Supplementary Figure S5). Next, the ratio of PKH26^high^ population was determined in sphere versus adherent condition which was defined as 1. In the controls, the ratio was from 1.3% to 2.5%, which rose 1.9 times; in the Rab27A ectopic groups, the ratio was from 1.4% to 3.4%, which rose 2.4 times (Figure 1J and 1N). As a contrast, the ratio of PKH26^high^ population in the RNAi controls was decreased from 7.3% to 2.1%, which reduced 3.5 times; in the Rab27A knocking down cells, the ratio value attenuated from 3.4% to 1.7%, which reduced 2 times (Figure 1K and 1M). Overall, these data provided further evidence validating the role of Rab27A in mediating positive effects on cCSCs.

### Rab27A overexpression redistributed cell cycle of cCSCs

**Figure 2:**
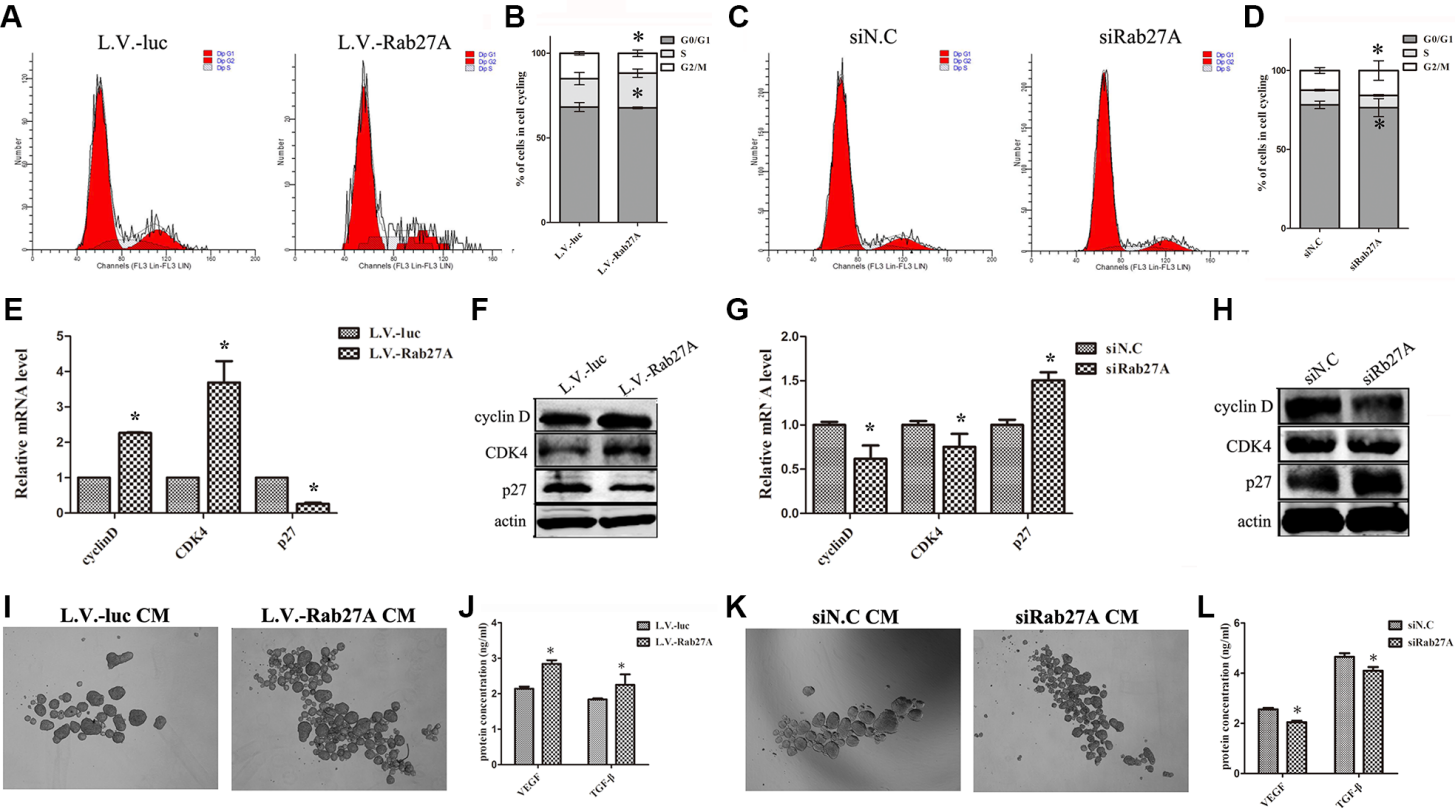
Rab27A overexpression redistributed cell cycle of cCSCs by promoting the secretion of VEGF and TGF-β *in vitro* (**A** and **C**) Cell cycle of HT29 spheroid cells was analyzed by FACS after ectopic Rab27A expression and knockdown. (**B** and **D**) Data of (A and C) were calculated as mean ± SD as detailed in Materials and Methods. Error bars denote the SD between triplicates (*p < 0.05). (**E** and **G**) qRT-PCR showing the expression level of cyclin D, CDK4 and p27 in HT29 spheroid cells. GAPDH served as a loading control. Error bars denote the SD between triplicates (*p < 0.05). (**F** and **H**) Western blot showing the expression level of cyclin D, CDK4 and p27 in HT29 spheroid cells. Actin served as a loading control. (**I** and **K**) Representative images of cCSCs spheres cultured in different conditioned medium. (**J** and **L**) ELISA analyses of VEGF and TGF-β protein in conditioned medium of sphere (*p < 0.05).

To further characterize the underlying mechanisms by Rab27A, we investigated the impact of Rab27A expression on cell cycle distribution by FACS. Rab27A overexpression accelerated the cell growth under spheroid condition, displaying a higher fraction of cells at S phase, but lower fractions at G0 and G1 phase (Figure 2A and 2B). After knocking down Rab27A, the growth of both HT29 spheroid cells was delayed (Figure 2C and 2D). Subsequently the changes of cell cycle related markers were determined, including cyclin D1, CDK4 and p27 in HT29 spheroids. The ectopic up- and down-regulations of Rab27A led to relevant fluctuations in cell cycle markers (Figure 2E–2H). These data confirmed the role of Rab27A in affecting the proliferation potential of HT29 spheroids cells *in vitro* with altered expressions of cyclin D, CDK4 and p27.

### Rab27A promoted the secretion of VEGF and TGF-β in cCSCs *in vitro*

As a small GTP protein, Rab27A is known to be responsible for extracellular secretion and exosome release. Therefore it was speculated that Rab27A may facilitate secretion of some factors, which are responsible for the expanded sphere. We observed the subcellular localization of Rab27A in HT29 cells after being transfected with pEGFP-Rab27A. As shown in Supplementary Figure S6, under confocal microscopy, Rab27A positive signals were eccentrically distributed in cytoplasm and apparently concentrated in the perinuclear area. This indicated that Rab27A may participate in HT29 cell secretions.

In order to test this, we performed sphere forming assay by transplanting the Rab27A modified spheroid culture supernatant. Compared with controls, the transplanted media plus with the supernatant from Rab27A overexpression sphere showed an obvious increase in HT29 sphere growth (Figure 2I), but the sphere growth was prohibited with the supernatant from Rab27A knocking-down sphere (Figure 2K). To gain insight into the molecular basis, we detected VEGF and TGF-β protein, which are famous for promoting the tumor growth, in conditioned media of spheroids by ELISA. Notably, the levels of both VEGF and TGF-β protein, in conditioned medium, was increased in Rab27A overexpression sphere cells (Figure 2J), whereas, the amount of VEGF and TGF-β in conditioned medium was limited in Rab27A knocking-down cells (Figure 2L). In either situation, both IL-4 and IL-6 were undetectable. These results suggested that Rab27A contributes to the growth of cCSCs, very likely by facilitating the secretion of VEGF and TGF-β.

### Rab27A promoted tumor formation and growth *in vivo*

One of key features of CSCs is efficient xenograft formation. To examine the effect of Rab27A on tumorigenicity of colon cancer cells, a gradient numbers of cell groups were inoculated into nude mice followed by weekly monitoring of tumor formation for 50 days. As shown in Table 1, when animals injected respectively with 1 × 10^3^, 1 × 10^4^ and 1 × 10^5^ cells overexpressed with Rab27A, all of the tumor incidences were higher than the controls transfected with L.V.-luc. At the same time, we found that Rab27A-T23N, which is a dominant negative mutant defective in GTP binding [35], prohibited tumor formation significantly. These data indicated that Rab27A increased the self-renewal potential of colon cancer cells *in vivo*.

**Table 1:** Incidence of tumors by HT29 cells in nude mice

Number of cells inoculated	1 × 10^5^	1 × 10^4^	1 × 10^3^
**L.V.-luc**	**5/8**	**3/8**	**1/8**
**L.V.-Rab27A**	7/8*	5/8*	2/8*
**L.V.Rab27A-T23N**	4/8*	1/8*	0*

* *p* ≤ 0.05 compared with HT29 cells transduced by luciferase (luc).

**Figure 3:**
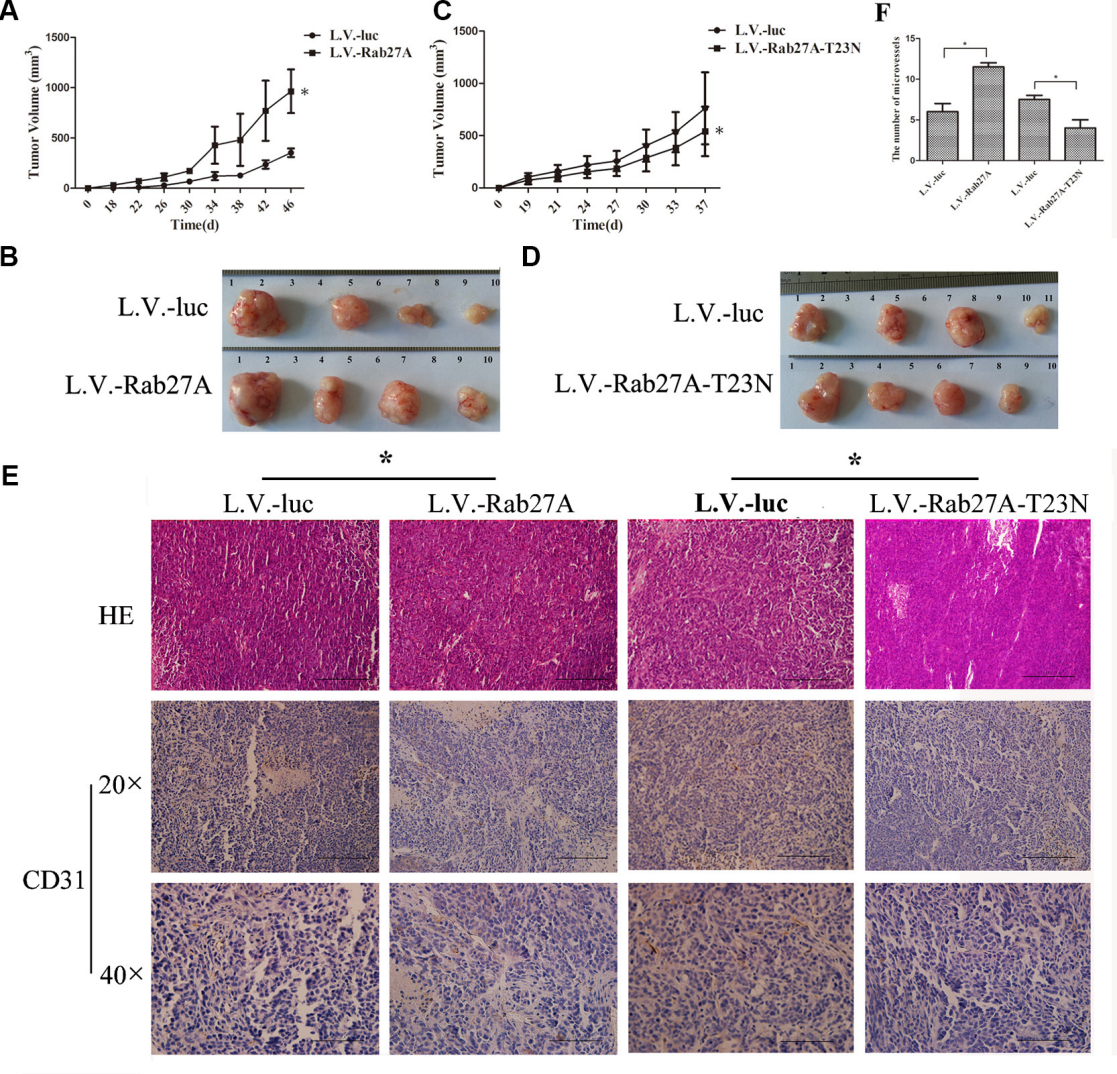
Rab27A promoted colon tumor growth *in vivo* (**A** and **C**) Female Balb/C nude mice were subcutaneously inoculated with HT29 sphere cells (1 × 10^4^). Tumor size was measured and tumor volume was calculated. Data was presented as mean ± SD (*n* = 8, *p < 0.05). (**B** and **D**) The mice were killed and the tumors were removed. Photograph of tumors developed in each group was shown. (**E** and **F**) Immunohistochemistry detection of microvessel density. The results of H&E staining and CD31 staining were presented (C), with a semiquantitative analysis of CD31 density performed by quantification of CD31-stained vessels in 20 field, at a magnification of 40 × (D). (*p < 0.05).

To further investigate whether Rab27A enhanced the growth of sphere cells of HT29 *in vivo*, we implanted 10^4^ sphere cells, stably expressing Rab27A and Rab27A-T23N, into nude mice. The Rab27A-expression sphere tumor grew more quickly than the control group (L.V.-luc) (Figure 3A and 3B). In contrast, the growth of sphere cell transduced with Rab27A-T23N was slower than control group (Figure 3C and 3D).

We observed that Rab27A over-expression enhanced the secretion of VEGF in HT29 sphere cells (Figure 2J), thus we went on to further explore the angiogenesis of the tumors by CD31 immunohistochemistry to measure the density of blood vessels. As shown in Figure 3E and 3F, microvessel density was elevated by Rab27A over-expression as compared with control group. In contrast, heterogenous expression of Rab27A-T23N depressed the angiogenesis of HT29 tumors. Overall, Rab27A promoted the sphere growth *in vitro* and *in vivo* by increasing the secretion of VEGF.

### p65 binds directly to the Rab27A promoter to regulate its expression

**Figure 4:**
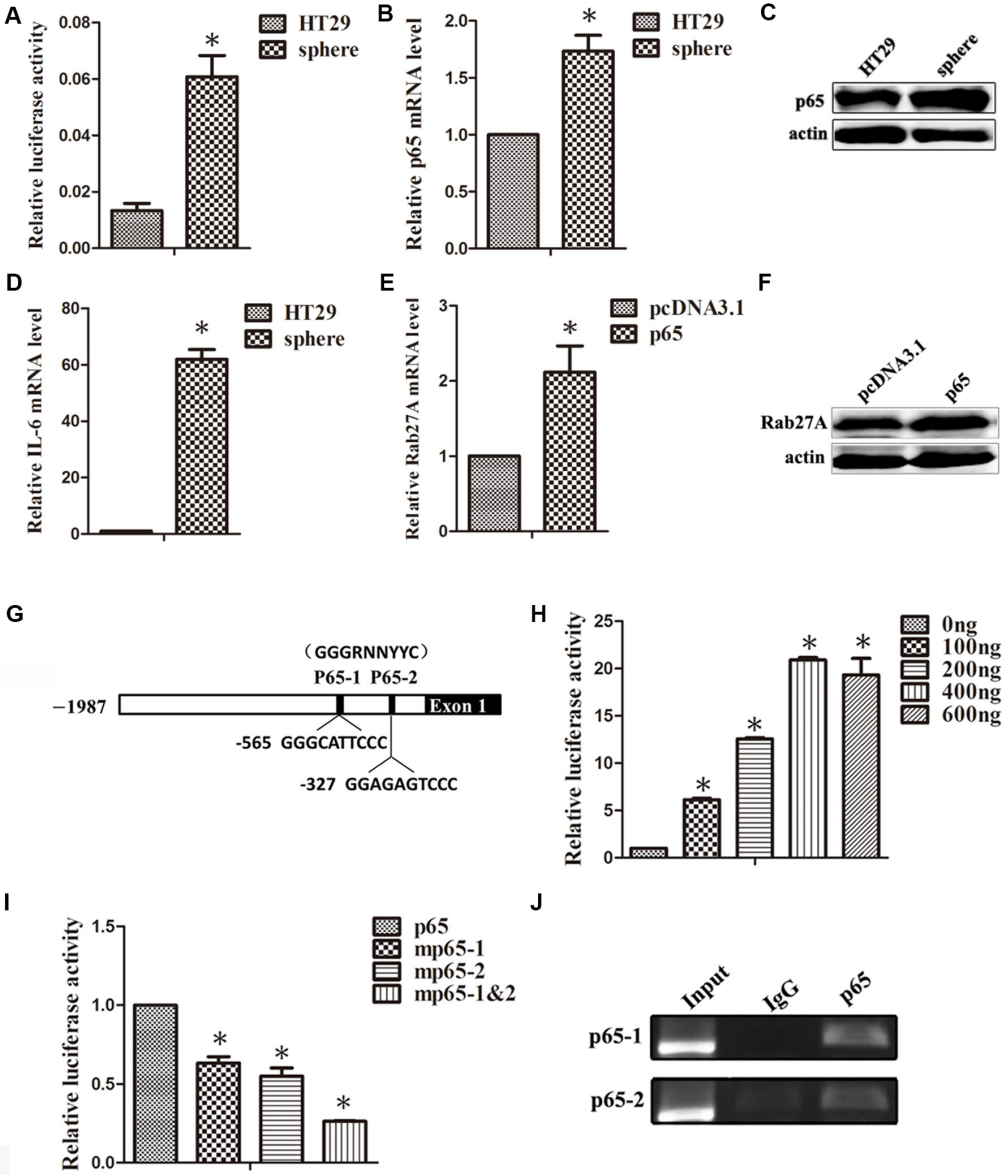
p65 binds directly to the Rab27A promoter to regulate its expression (**A**) The activation of NF-κB signaling pathway was detected in HT29 sphere cells, transiently expressed with NF-κB luciferase reporter plasmid. Error bars denote the SD between triplicates (*p < 0.05). (**B** and **C**) qRT-PCR (B) and Western blot (C) analysis of the expression for p65 in HT29 adherent cells and sphere cells. (**D**) qRT-PCR analysis of the mRNA expression for IL-6, the target molecule of p65, in HT29 adherent cells and sphere cells. (**E** and **F**) qRT-PCR (E) and Western blot (F) analysis of the expression for Rab27A in HT29 cells transfected with p65 for 48 h. (**G**) The schematic of p65-binding-element of Rab27A promoter. (**H**) The plasmid of Rab27A promoter and different concentrations of p65 were introduced into HT29 cells together for 24 h. The luciferase activity was measured and normalized according to *Renilla* luciferase activity. Error bars denote the SD between triplicates (*p < 0.05). (**I**) Wild-type and p65-bingding-element-mutated Rab27A promoter were transiently expressed in HT29 cells. The relative luciferase activity is present as the means ± standard error from three independent experiments. (**J**) Chromatin immunoprecipitation (ChIP) of p65 and Rab27A promoter.

It was previously reported that NF-κB signaling was able to mediate cell transformation, especially cancer stem cell formations. It's intriguing to observe the activation status of NF-κB in HT29 sphere cells. Firstly, HT29 transiently expressed with NF-κB luciferase reporter plasmid was cultured in suspension. 5 days later, the reporter activity of NF-κB was measured and shown to be increased (Figure 4A). At the same time, the expression of p65 was elevated in HT29 sphere cells (Figure 4B and 4C). We also observed that the expression of IL-6, a direct target gene of NF-κB, was up-regulated (Figure 4D). More importantly, the expression of Rab27A at both mRNA and protein levels were indeed induced in response to p65 overexpression (Figure 4E and 4F). These results supported that the up-regulated expression of Rab27A in HT29 sphere may be induced via the increased expression of p65.

To further clarify the molecular mechanism of Rab27A up-regulation in HT29 sphere cells, we constructed the Rab27A promoter vector within the upstream −2000 to +1 regulatory region, consisting of two p65 consensus binding sites (Figure 4G). The Rab27A-Luc reporter assay disclosed that the luciferase activity intensified following p65 overexpression in a dose-dependent manner, and reached a saturation value at 400 ng (Figure 4H).

Subsequently, in order to locate p65 direct binding sites, we introduced point mutations into the p65-1 and p65-2 with TC in replacement of GA. As shown in Figure 9C, compared to wild type, the mutant reporter driving luciferase activity declined, especially when two mutations concurred together (Figure 4I). Chromatin- immunoprecipitation (ChIP) assay was subsequently used. Compared with control IgG, p65 antibody displayed a more specific signal on Rab27A promoter (Figure 4J). To this end, we conclude that the higher Rab27A level in colon sphere was directly driven by inflammation-related NF-κB signals.

## DISCUSSION

In recent years, more and more evidences demonstrated that cancers may arise from rare cancer stem cells (CSCs). Colon cancer is one of the solid tumors comprising of a heterogeneous groups of cells [1–3], among which specific cancer stem cells can be marked, by CD133^+^ or CD44^+^, or the increased intensity of PKH26 dye labeling. Previous studies reported that canonical Wnt signaling activation, especially nuclear β-catenin importing is one of the common pathways in tumor initiation, as well as in cancer stem cell transformation [21], whereas NF-κB activation triggered by inflammation related signals present in tumor environment may induce dedifferentiation of colon epithelia by inducing Wnt activation [23, 25]. In a way, CSC is endowed with the ability to influence the surrounding environment, which may be beneficial to its survival and metastasis. Rab27A is the key protein for intracellular secretion [26–29], and was reported to promote tumor progression [30–34]. Putatively Rab27A mediated autocrine and paracrine effects may facilitate the cross-talk between CSC and environment. Therefore this study revealed for the first time that the increased paracrine activity in cCSCs mediated by Rab27A expression is one of key intrinsic features, contributing to the enhanced cCSC tumorigenicity.

Using colon cell lines, we set up suspension spheroid culture condition and measured cCSC specific parameters, including CD44^+^, PKH26 dye retaining capability. We detected multiple stem marker genes and differentiated genes through 5 rounds of sphere propagations. All these assays provided substantial evidence in identifying cCSC features. Through ectopic expression and RNAi knocking-down of Rab27A, these cCSC assays substantiated the growth promoting effects of Rab27A. Our results from supernatant transferring in spheroid propagations, along with ELISA measurements of cytokines, such as VEGF and TGF-β, are in supportive of Rab27A mediated autocrine or paracrine effects.

Quiescent was the defining characteristic of a stem cell, and was to develop fully functioning tissues via proliferation and differentiation. Here we found that the proliferation of HT29 spheres was significantly decreased, comparing with adherent HT29 cells, by FACS (Supplementary Figure S7). This data indicated that cCSCs also had a higher proportion of quiescent cells, those in attesting stage in the cell cycle. Our results shown that overexpression of Rab27A promoted the proliferation of cCSCs. But we still needed to discuss in depth whether Rab27A effected the differentiation of cCSCs.

It is notable that high levels of Rab27A were detected in both well differentiated normal colon epithelia cells and cancer stem cells, indicating that it is not a specific cancer stem cell marker, but may rather serve a complementary role in promoting the exocrine activity in cancer stem cells. Regarding the particular role of elevated levels of VEGF in sphere culture media, it may provide self-growth signals, and may also have nearby angiogenesis promoting effects as found in our *in-vivo* xenograft model. However, since we did not set up the homogenous mice transplant model, it was difficult for us to observe TGF-β mediated immune suppressive effects. Besides, in the cCSCs co-cultured with transferred supernatant, regardless of VECF's effects, TGF-β may also directly induce the Epithelial-Mesenchymal Transition (EMT) process, which we have not clarified at this stage. As other studies have shown, exosome secretion is also involved in tumor development especially in distant metastasis [32, 34, 36]. Future experiment in this area will be required in order to fully investigate the influence of Rab27A on metastasis.

Finally regarding the increased expression of Rab27A in cCSC, we found that one of NF-κB signals, p65, had direct specific binding on putative cis-elements in Rab27A promoter which occurred mainly in heightened levels of Rab27A. Overall, our experiments defined a novel pathway linking environment inflammation signals with cCSC survival and growth effects via amplified autocrine mechanisms.

## MATERIALS AND METHODS

### Immunohistochemistry on tissue microarrays and xenograft tumors

Clinical data and primary colon carcinoma samples and adjacent histologically normal colon tissues were purchased from Shanghai Outdo Biotech Co. An informed consent was obtained from every patient according to the company and the study was approved by the ethical committee of the appropriate institutions. Tissue microarrays and xenograft tumor slides were incubated with primary antibody anti- Rab27A (1:150; Sigma) or anti-CD31 (Abcam) for 24 h at 4°C. Anti-rabbit peroxidase-conjugated secondary antibodies were applied. Whole tissue sections were examined.

Immunohistochemical analysis indicated that the Rab27A positive staining cells were mainly localized in the cytoplasm, and were brown. The percentage of positive cells was calculated in 10 randomly-selected fields, at a magnification of 200×. The specimen, the percentage of positive cells of which was over than 20%, was defined as Rab27A positive expression.

### Cell lines and tumor sphere culture

Colon cancer cell lines HT29, Caco-2, DLD-1, SW480 and SW620 were purchased from Cell Bank of Type Culture Collection of the Chinese Academy of Sciences (Shanghai Institute of Cell Biology, Chinese Academy of Sciences), and were maintained in complete growth medium as recommended by the manufacturer.

For tumor sphere formation assay, single cells were plated in ultralow attachment plates (Corning) at a density of 1000 viable cells/mL in primary culture and in passages. Cells were grown in a serum-free DMEM/F12 (Gibco), supplemented with B27 (Invitrogen), 20 ng/mL human recombinant epidermal growth factor (hEGF; Sigma) and 10 ng/mL basic fibroblastic growth factor (bFGF; Sigma), 0.4% bovine serum albumin (BSA; Sigma), and 4 mg/mL Insulin (Sigma). Cells were cultured for 4–8 days and the tumor sphere with a diameter of ≥ 50 mm were counted and represented graphically.

### PKH26 staining and flow cytometry assay

PKH26 staining was performed as described earlier [37]. HT29 cells were stained with PKH26 dye (Sigma; 1:250) as per manufactures instruction. The staining was stopped by adding serum to the cell suspension. Cells were cultured in adherent and sphere condition respectively. After 5 days, cells were digested by Accutase into single cells and PKH26^high^ cells were analysed by FACS.

Cells were then incubated with primary antibody CD44-FITC (dilution 1/100 for 10^6^ cells/100 ml, BD Biosciences) for 30 min, on ice, in RPMI 1640 (GIBCO) with 3% bovine serum albumin (BSA), followed by washing in PBS with 3% BSA. Analysis was performed using a FACS Vantage SE flow cytometer (Becton& Dickinson).

### Tumorigenicity assays in nude mice

Indicated numbers of single cells dissociated from spheres were suspended in 200 μl of PBS and Matrigel (1:1) (Becton Dickinson) and injected subcutaneously into 6-week-old female Balb/c nude mice (Shanghai, China). The incidence and size of subcutaneous tumors were recorded every 3 days. Tumor volumes were calculated as Volume (mm^3^) = Length (mm) × Width (mm)^2^/2. Mice were sacrificed by cervical dislocation. Tumor xenografts were harvested and embedded into paraffin. Tumor tissues characteristics were determined by microscopic inspection after hematoxylin and eosin (H&E) staining. All animal procedures were carried out in accordance to the ethical guidelines established by the Fourth Military Medical University under an approved animal protocol.

### Luciferase activity assay

To detect NF-κB activity in colosphere, HT29 cells were transfected with NF-κB-luc reporter vector and Renilla-luc control vector (Promega). Twenty-four hours after transfection, cells were digested with Accutase for about 5 mins, then the single cells were cultured to generate colospheres and growth in adherent condition. After 5 days, the colosphere were lysed and luciferase activity was measured using Dual Luciferase kit (Promega) according to manufacturer's instructions.

In order to detect the binding of p65 to the promoter region of Rab27A, four HT29 cells lines were prepared. All cell lines were transfected with p65, along with Renilla-luc as a control vector. pGL-3-Rab27A, pGL-3-Rab27A-1, pGL-3-Rab27A-2, and pGL-3-Rab27A-1&2 were then each transfected into one cell line. After 24 hours, cells were lysed and assayed for luciferase activity.

### Chromatin immunoprecipitation (ChIP)

ChIP assays were performed following the protocol as described previously [38, 39] to investigate whether p65 binds *in situ* to the *Rab27A* promoter. HT29 cells were cultured on dishes and were transfected with p65 for 24 h. Cells were then fixed with 1% formaldehyde for 15 min, followed by a 5-min treatment with 1.25 M glycine to quench the reaction. Nuclear extracts were sonicated to shear DNA into 500-bp fragments. Precleared lysates were subjected to overnight immunoprecipitation with 2 ug/ml of rabbit anti-p65 antibody or normal rabbit IgG (negative control). DNA samples were recovered after phenol/chloroform extraction and isopropanol precipitation. A volume equal to that of the final precipitate was used for PCR amplification with specific primers (Supplementary Table S1), which gave rise to a Rab27A promoter region containing a putative p65 binding site.

### Statistics

All *in vitro* experiments were performed either in triplicate or in reduplicate. Results are presented as mean ± standard deviation (SD). Comparison among groups was performed by independent sample *T-test* or Bonferroni's multiple-comparison *T-test*. Statistical analyses were performed using IBM SPSS software.

Additional Materials and Methods are available in the Supplementary Materials

## SUPPLEMENTARY MATERIALS FIGURES AND TABLES


