# Supplementary material for: Rab27A mediated by NF-κB promotes the stemness of colon cancer cells via up-regulation of cytokine secretion
Source: Oncotarget. 2016 Aug 20;7(39):63342–51. doi: 10.18632/oncotarget.11454 (PMC5325368; doi:10.18632/oncotarget.11454)
Supplement: Supplementary file 1 [file oncotarget-07-63342-s001.pdf]

# Rab27A mediated by NF-κB promotes the stemness of colon cancer cells via up-regulation of cytokine secretion

## Supplementary Materials

## MATERIALS AND METHODS

### Sphere culture

For serial passage, sphere were collected by gentle centrifugation (800 rpm, 10 min) and dissociated by Accutase (Sigma) for 15 min to obtain single-cell suspensions. Single cells were then cultured to generate spheres of the next generation.

For condition medium culture, we extracted supernatant from HT29 sphere and Rab27A overexpression sphere at day 5, then used a 1:1 ratio of sphere medium and sphere supernatant to further culture HT29 cells. After 5 days, spheres with a diameter of ≥ 50 mm were counted and represented graphically.

### Plasmid construction, virus packaging, and infection

The entire open reading frame of the human *Rab27A* gene (Gene ID 5873) was amplified from 293T cells by Reverse Transcription-PCR using the gene-specific primers, and then fused in-frame into the pEGFP-C2 vector (Clontech, Mountain View, CA), establishing pEGFP-Rab27A.

For the construction of luciferase reporter, the complete DNA sequence of Rab27A promoter and the mutated p65 binding sites were amplified, and was subsequently cloned to the pGL3 basic luciferase reporter vector (Promega), named pGL-3-Rab27A, pGL-3-Rab27A-mp65-1, pGL-3-Rab27A-mp65-2, respectively.

To establish the stable cell lines overexpressing Rab27A and Rab27A-T23N (dominant negative mutants defective in GTP binding), the cDNA of Rab27A and Rab27A-T23N was subcloned in-frame into the multiple cloning site of pENTR$^{TM}$3C vector (Invitrogen$^{TM}$). Rab27A was subcloned into Destination vector by LR recombination reaction. All constructs were confirmed using enzyme digestion and DNA sequencing. Detailed primers information used in this study are listed in Supplementary table 1. Virus packaging was performed as recommended by the manufacturer. The lentivirus was infected into the colon cancer cells with Polybrene (Sigma). The infected cells were selected in blasticidine (Sigma).

### qRT-PCR

Total RNA was extracted by the TRIzol method according to the manufacturer's instructions. cDNA was synthesized from 0.5–1 mg of total RNA using the Superscript II reverse transcriptase (Invitrogen). Real-time quantitative reverse transcription PCR (qRT-PCR) was performed. SYBR green II (Invitrogen) was used to detect PCR products. All reactions were done in a 10 μl reaction volume in triplicate. PCR amplification consisted of 10 min of an initial denaturation step at 95°C, followed by 45 cycles of PCR at 95°C for 30 s,56°C for 30 s and 72°C for 15 s. Results were analyzed using the comparative ΔΔCt method. Data are presented as the fold difference in gene expression normalized to the housekeeping gene human GAPDH and relative to a relevant reference sample.Detailed primers information used in this study are listed in Supplementary Table S1.

### Western blot

Havested cells were lysed in a radio immunoprecipitation assay (RIPA) lysis buffer, and the lysate was cleared by the centrifugation at 12,000 rpm for 20 min. The supernatant was collected as total proteins. Twelve percent sodium dodecyl sulfate polyacrylamide gel electrophoresis (SDS- PAGE) was used for separating total proteins into equal amount (50 μg each) which were then transferred to polyvinylidene fluoride (NC) membranes (Invitrogen, USA). The membranes were incubated with antibodies raised against Rab27A (1:500; Sigma-Aldrich), p65 (1:800; Cell Signaling), Cyclin D (1:500; Santa Cruz), CDK4 (1:500; Santa Cruz), p27 (1:500; Santa Cruz) and β-actin at 4°C overnight. GAPDH was used as an internal control. The secondary antibodies anti-rabbit IgG or anti-mouse IgG were both from Odyssey. Western blot bands were obtained using Imaging System, and the protein density was quantified with Odyssey v1.2 software (LI-COR Biosciences, USA).

### Cell cycle analysis

For the analysis of cell cycle distribution, cells were starved in a serum free culture for 24 hours, and

then incubated in sphere culture medium for 5 days. Cells were harvested and treated as recommended by the manufacturer for RT-PCR and Western blot. For flow cytometry assay, cells were digested by trypsinization, washed with PBS and exposed to DNA Prep Lyse for 1 minute, followed by incubation with DNA Prep Stain for 15 minutes at room temperature in the dark. Cellular DNA content was monitored on a FACS Vantage SE flow cytometer (Becton&Dickinson).

### Measurement of VEGF and TGF-β by ELISA

The concentration of VEGF and TGF-βreleased to the supernatant of colosphere was measured via VEGF and TGF-βELISA assays (Neobioscience Technology) according to manufacturer's instructions.

### Transfection and confocal microscopy

To characterize the localization Rab27A in colon cancer cells, HT29 cells were transiently transfected with pEGFP-Rab27A using lipofectamine™ 2000 (Invitrogen, Carlsbad, CA). After 36 hours, cells were fixed in cold methanol for 10 min. Nuclear DNA was stained with 4,6-diamidino-2- phenylindole. For confocal microscopy with fixed cells, optical sections were scanned at 0.1- to 0.2-Am intervals as needed. Z-stack images were then formed by maximal projection.

## RESULTS

### The identification of the stemness of HT29 sphere

As the number of colon cancer stem cells is one of indicator for tumor malignancy, it'll be interesting to observe whether Rab27A affects CSC formation. Therefore we set up the colon sphere suspension culturing by using cell lines. After 5-days, colospheres derived from the cell lines were formed, including HT29, Caco-2, DLD-1, SW480, SW620 and HCT116, differing with each other in morphologies. The HT29 cells could form classical spheres in serum free culture (Supplementary Figure S1).

Multiple serial passaging of sphere was performed to predict the self-renewal potential of spheroid cells. We passaged HT29 spheres for 5 times. And we found that various colon cancer stem cell markers, such as CD133, Oct4, EpCAM, Bmi-1, Musashi-1, ABCG2, CD44, Nanog, lin28a and Lactase, tended to be maintained at higher levels in passaging of HT29 sphere cells than in adherent cells (Supplementary Figure S2A and S2B). In contrast, the expression of level of the differentiation markers, such as CK20, CDX1 and CDX2, were drastically decreased in passaged HT29 sphere cells (Supplementary Figure S2C). These data indicated that the sphere culture system could be used for study the stemness of cancer cells. In addition, we observed that the expression of stem cell markers and Rab27A was decreased in passage 5. We speculated that the sphere culture system here was suitable for screening and enriching CSCs, but not appropriate to culture CSCs in the long term. The stem cells might start differentiating after 5 passages.

### The proportion of PKH26$^{high}$ cells was increased in HT29 sphere cells

PKH26 is a dye which binds membrane phospholipids, thus conferring a bright red fluorescence. PKH26 staining may represent an unconventional proliferative assay and a surrogate marker of stemness of breast and colon cancer. In fact, the dye segregates during each cell division, and a progressive decrease of fluorescence intensity in daughter cells was observed, the stem-like cells are monitored by FACS based on their brighter colors due to retarded multiplication. Here, HT29 cells after being labeled with PKH26 and cultured in suspension and adherent conditions, 5 days later, PKH26 staining was taken by immunofluorescence microscope (Supplementary Figure S4A), and the proportion of PKH26high cells were calculated by FACS. Based on the SFE of HT29 cells, as few as 1 to 3% of the total cell population with the most intense fluorescence was defined as PKH26$^{high}$ cells. Compared with adherent HT29 cells, the ratio of PKH26$^{high}$ population was at 1.6%, in colon sphere condition, up to 9.1% was determined as the PKH26$^{high}$ (SupplementaryFigure S4B). This data highly indicated that colon sphere enriches more stem-like cells.

Allthe above detection methods indicated that colon sphere was indeed enriched with higher stem cell proportions. In contrast with CD44$^{+}$ markers detection, PKH26 staining method is more stringent for stem cell determination. Altogether, the above results demonstrated that our sphere suspension culture system could be used to isolate and enrich cCSCs. The ratio of CD44$^{+}$ and PKH26$^{high}$ were suitable stemness markers of cCSCs.

**Supplementary Table S1: Primers and sequences used in the experiment**

| Name | Forward (5′–3′) | Reverse (5′–3′) |
|---|---|---|
| Rab27AcDNA | CGCGGATCCATGTCTGATGGAGATTATG | CCCAAGCTTTCAACAGCCACATGCCCCTT |
| Rab27A T23NcDNA | TGTAGGGAAGAACAGTGTACTTTAC | |
| siRab27A | CGGAUCAGUUAAGUGAAGAAA | UUUCUUCACUUAACUGAUCCG |
| NC siRNA | UUCUCCGAACGUGUCACGUTT | ACGUGACACGUUCGGAGAATT |
| Rab27Apromotor | CGGGGTACCCTTGTTTACTAATAGGGTGCC | CCGCTCGAGCTGGCCCCACATCACTT |
| p65-1mutant site | CAGGAATCCTGGGCATGACCTTGCCCCGGAAACG | |
| p65-2mutant site | GGTGAATAGGGGAGAGGACCCAAACGACACCTCGCC | |
| CHIP p65-1 | CTCCGTTTGCCATTTCCTCC | AGGGTGTTTTCCACGGCATC |
| CHIP p65-2 | GTGTCCAGGCCGACCTAA | ACAAACTGCACCAAGGACCG |
| Rab27A RT-PCR | TGGAGGACCAGAGAGTAGTGAAA | AGTTTCAAAGTAGGGGATTCCA |
| p65 | GGGAGCTGGGGAAACTCAAA | CTCCAGCCTGCTTCTGTCTC |
| CD44 | AAAGGAGCAGCACTTCAGGA | TGTGTCTTGGTCTCTGGTAGC |
| CD133 | GCATTGGCATCTTCTATGGTT | GCCTTGTCCTTGGTAGTGT |
| Oct-4 | AAGCAGAAGAGGATCACC | GCAGCTTACACATGTTCTT |
| Nanog | AAAGAGGTCTCGTATTTGCTGC | AACACTCGGTGAAATCAGGGTA |
| Lin28a | GTGCGGGCATCTGTAAGTGG | CACTGCCTCACCCTCCTTCA |
| EpCAM | CTGCCAAATGTTTGGTGATG | AAAGCCCATCATTGTTCTGG |
| ABCG2 | AGCTGCAAGGAAAGATCCAA | CAGAGTGCCCATCACAACAT |
| Musashi-1 | GGGGTCAGCAGTTACATCAG | TACCCATTGGTGAAGGCTGT |
| Bmi-1 | GGTATTCCCTCCACCTCTTC | GCTGTTGCTGGTTCCATTCA |
| Lactase | TTTGGGGACCGTGTGAAG | CCACTCCTGGGTCAGAGAT |
| CK20 | CCTCAAAAAGGAGCATCAGG | ATGATGACGCCAAGGTTCA |
| CDX1 | TGAACGGCAGGTGAAGATCTG | GCTGTTTCTTCTTGTTCACTTTGC |
| CDX2 | CAGCCAAGTGAAAACCAGGA | GGCTAGCTCGGCTTTCCT |
| cyclin D | TGTGCATCTACACCGACAAC | AGGAAGTGTTCAATGAAATCGT |
| CDK4 | TTGCATCGTTCACCGAGATC | CTGGTAGCTGTAGATTCTGGC |
| p27 | GCTAACTCTGAGGACACGCA | TAGAAGAATCGTCGGTTGCAGG |
| GAPDH | GACCTGACCTGCCGTCTA | AGGAGTGGGTGTCGCTGT |

NOTE. Primers and sequences used in the experiment. The restricted endonuclease sites and mutant sites are underlined. N.C. is negative control.

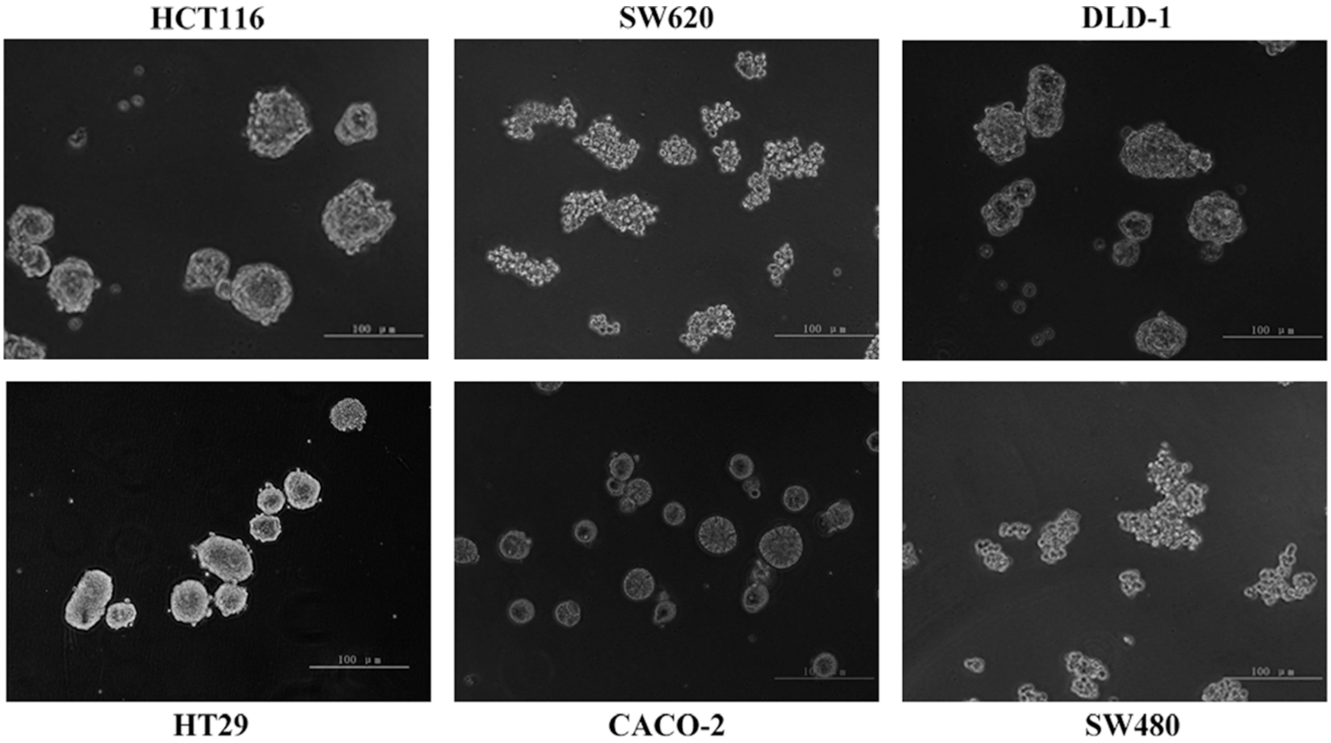


**Supplementary Figure S1: Representative images of spheres from 6 different colon cancer cell lines taken by optical microscope.**

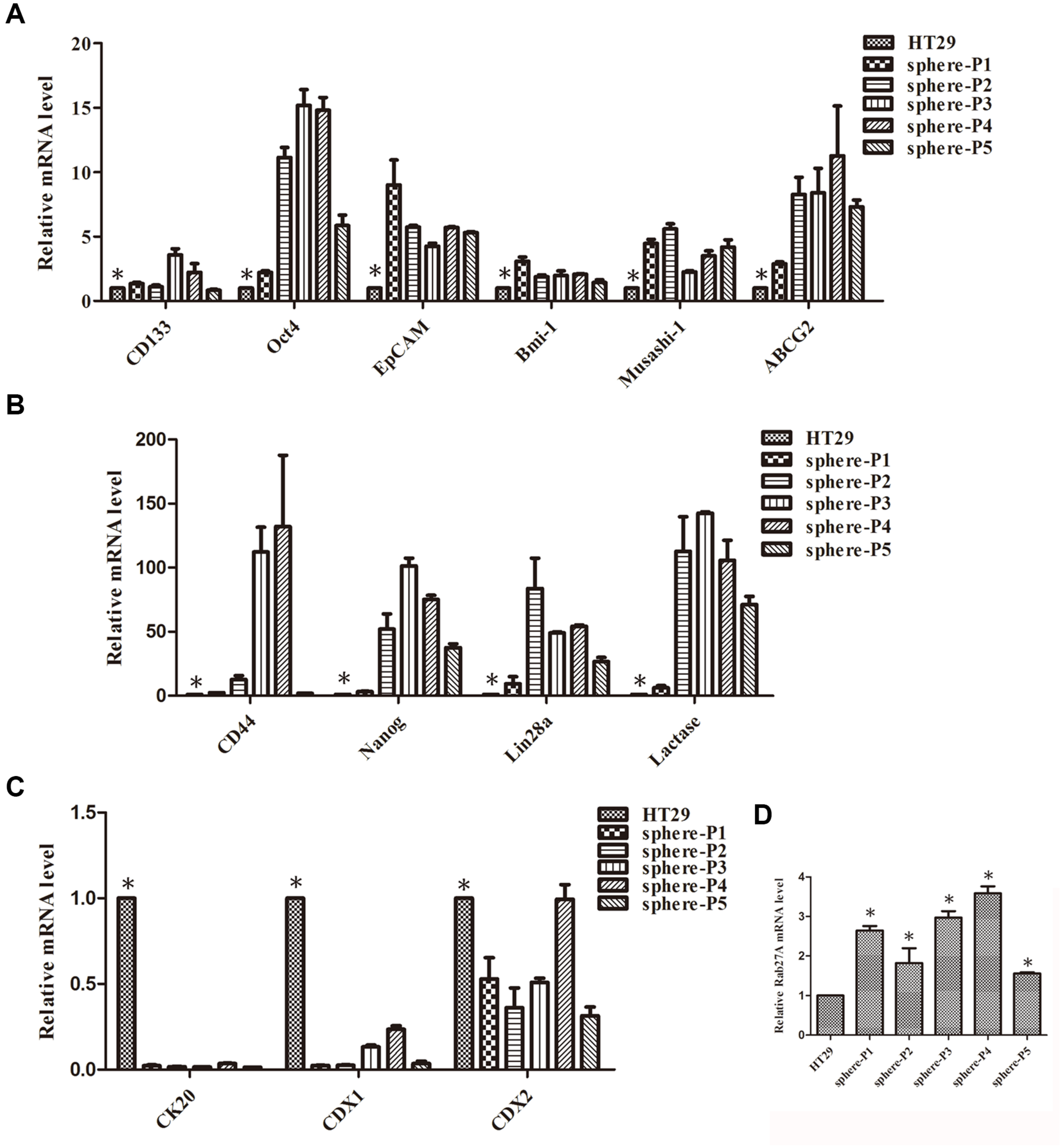


**Supplementary Figure S2: qRT-PCR showing the markers of stemness was up-regulated.** (**A** and **B**),the markers of differentiation was down-regulated (**C**) and the expression of Rab27A was increased (**D**) in serial spheres of HT29. Error bars denote the SD between triplicates (*$p < 0.05$).

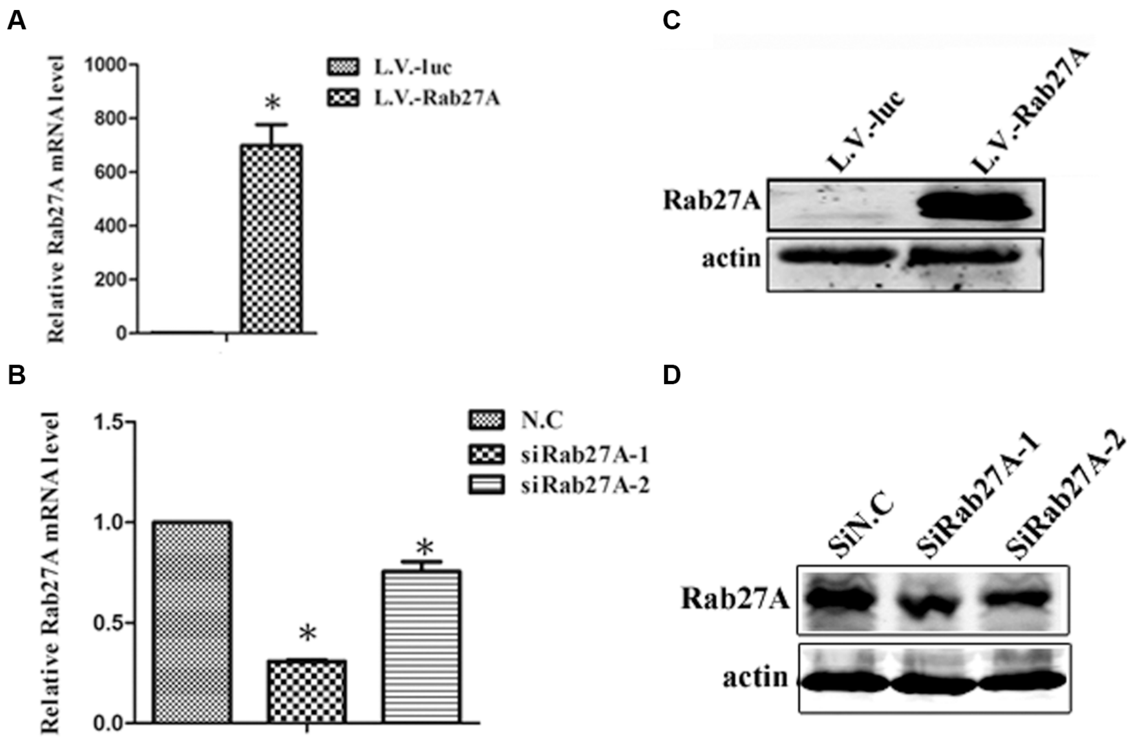


**Supplementary Figure S3:** (**A** and **B**) RT-PCR and (**C** and **D**) Western Blot analysis of mRNA and protein level of Rab27A after overexpression and interference respectively. Error bars denote the SD between triplicates (*$p < 0.05$).

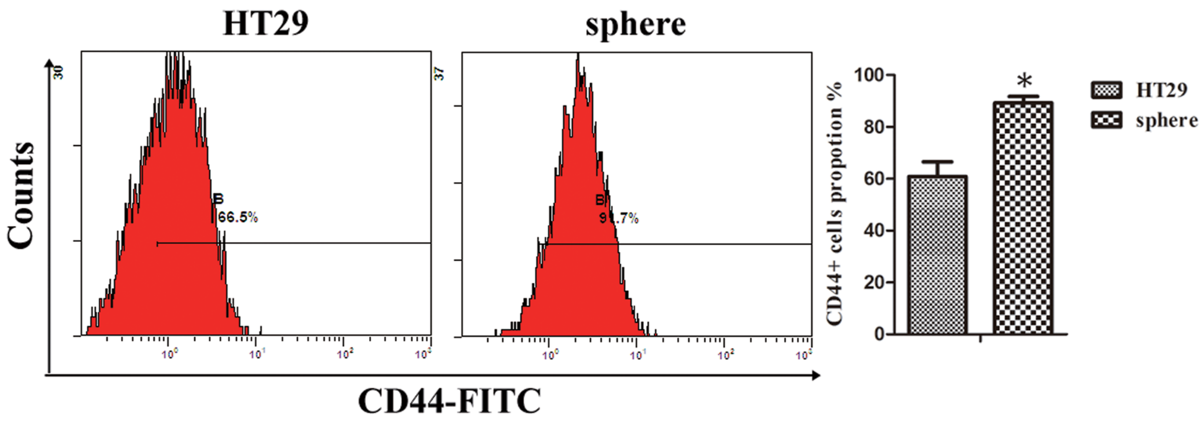


**Supplementary Figure S4: FACS showing CD44$^{+}$ levels in HT29 adherent cells and spherecells.** Error bars denote the SD between triplicates (*$p < 0.05$).

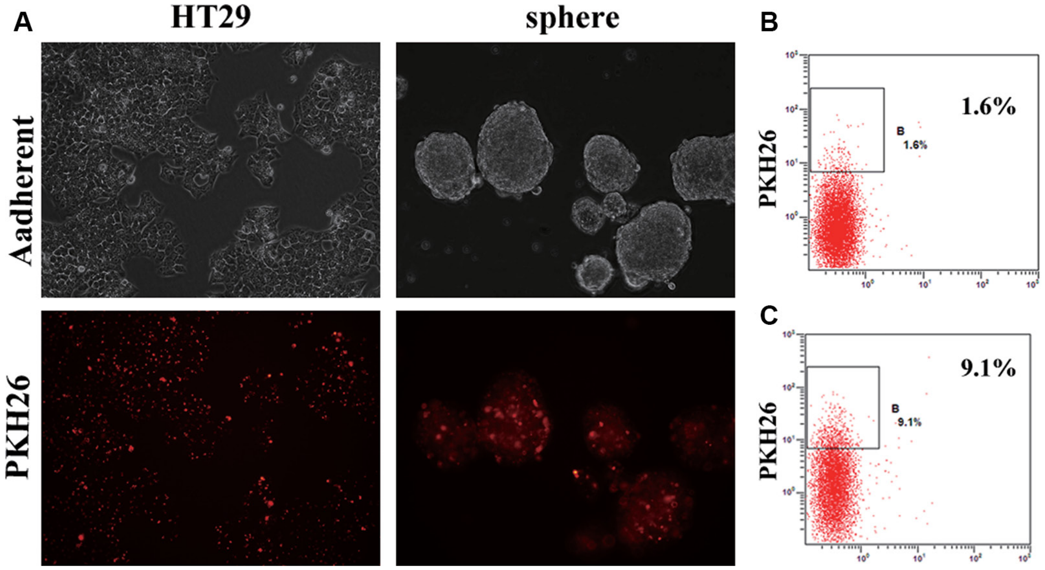


**Supplementary Figure S5: PKH26 staining of HT29 adherent cells and sphere cells.** (**A**) PKH26 staining was taken by immunofluorescence microscope. (**B**) The proportion of PKH26$^{high}$ cells in HT29 adherent cells was analysed by flow cytometry. (**C**) The proportion of PKH26$^{high}$ cells in HT29 sphere cells analysed by flow cytometry.

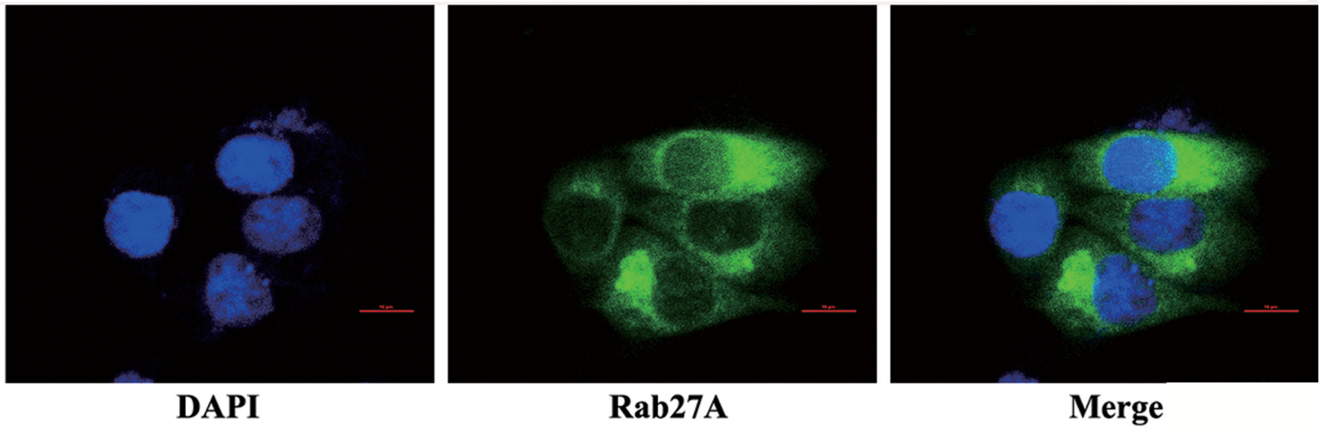


**Supplementary Figure S6: HT29 cells were transiently transfected with pEGFP-Rab27A.** After 36 hours, subcellular localization of Rab27A in HT29 cells was detected by immunofluorescence confocal microscopy.

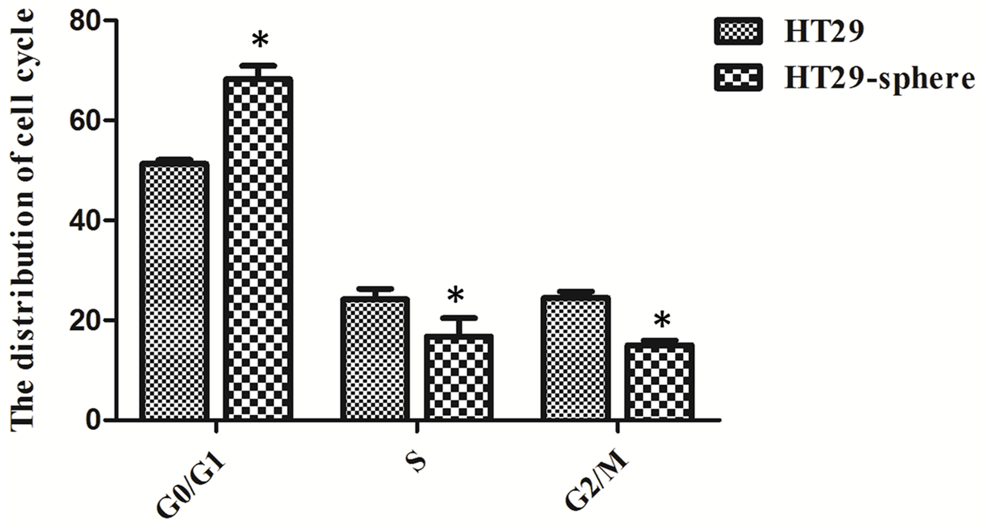


**Supplementary Figure S7: HT29 cells were transiently transfected with pEGFP-Rab27A.** Error bars denote the SD between triplicates (*$p$ < 0.05).
